# Reconstruction of Beagle Hemi-Mandibular Defects with Allogenic Mandibular Scaffolds and Autologous Mesenchymal Stem Cells

**DOI:** 10.1371/journal.pone.0105733

**Published:** 2014-08-25

**Authors:** ChangKui Liu, XinYing Tan, JinChao Luo, HuaWei Liu, Min Hu, Wen Yue

**Affiliations:** 1 Department of stomatology, General Hospital of the PLA, Beijing, China; 2 Department of Stem Cell and Regenerative Medicine Lab, Beijing Institute of Transfusion Medicine, Beijing, China; 3 Department of Stomatology, The 451th hospital of the People's Libration Army, Xi’an, China; Instituto Butantan, Brazil

## Abstract

**Objective:**

Massive bone allografts are frequently used in orthopedic reconstructive surgery, but carry a high failure rate of approximately 25%. We tested whether treatment of graft with mesenchymal stem cells (MSCs) can increase the integration of massive allografts (hemi-mandible) in a large animal model.

**Methods:**

Thirty beagle dogs received surgical left-sided hemi-mandibular defects, and then divided into two equal groups. Bony defects of the control group were reconstructed using allografts only. Those of the experimental group were reconstructed using allogenic mandibular scaffold-loaded autologous MSCs. Beagles from each group were killed at4 (n = 4), 12 (n = 4), 24 (n = 4) or 48 weeks (n = 3) postoperatively. CT and micro-CT scans, histological analyses and the bone mineral density (BMD) of transplants were used to evaluate defect reconstruction outcomes.

**Results:**

Gross and CT examinations showed that the autologous bone grafts had healed in both groups. At 48 weeks, the allogenic mandibular scaffolds of the experimental group had been completely replaced by new bone, which has a smaller surface area to that of the original allogenic scaffold, whereas the scaffold in control dogs remained the same size as the original allogenic scaffold throughout. At 12 weeks, the BMD of the experimental group was significantly higher than the control group (p<0.05), and all micro-architectural parameters were significantly different between groups (p<0.05). Histological analyses showed almost all transplanted allogeneic bone was replaced by new bone, principally fibrous ossification, in the experimental group, which differed from the control group where little new bone formed.

**Conclusions:**

Our study demonstrated the feasibility of MSC-loaded allogenic mandibular scaffolds for the reconstruction of hemi-mandibular defects. Further studies are needed to test whether these results can be surpassed by the use of allogenic mandibular scaffolds loaded with a combination of MSCs and osteoinductive growth factors.

## Introduction

Mandibular defects can be caused by ablative surgery for oral and maxillofacial tumors, trauma, infection, or congenital deformities. The reconstruction of large mandibular defects is a highly challenging task for oral and maxillofacial surgeons. Despite the many reconstructive methods available, autologous grafts are considered to be the “gold standard” because of their advantages of osteogenesis, osteoinduction, and osteoconduction. The iliac crest is the most frequently chosen donor site because it provides easy access to good-quality cancellous autografts in appreciable numbers. However, harvesting autologous bone from the iliac crest lengthens the overall surgical procedure and is usually complicated by hematoma formation, pelvic instability, nerve injury, residual pain, and cosmetic disadvantages [1]. Also, the shape of the mandible reconstructed by autogenous bone grafts is poor. Hence, a better method for mandibular reconstruction is required [2].

As the number of bone banks has increased, so has the number of bone allografts used in reconstructive surgery to replace missing bone parts (e.g., critical size defects) [3]. Most massive allografts have long-term success, but 25% of reconstructions fail because of infection, fracture, or nonunion [4]–[6]. In humans, graft union at the host bone is a slow process. The effectiveness of this procedure is dependent upon the healing time and type of graft integration. The larger the amount of bone to be replaced, the more difficult is the integration. This process may involve only 20% of the graft over 5 years, as shown by studies on retrieved allografts [7]. The allograft is also far from being an “ideal” option for bone reconstruction because of the risk of triggering host immune responses and their lack of osteogenic capacity.

To overcome these shortcomings in bone grafts, scientists have attempted to develop a bone construct using the “traditional triad” of tissue engineering (including sufficient osteocompetent cell transfer), structured scaffolding (to maintain space and provide osteoconduction), and the application of miscellaneous growth factors (which can induce adjacent mesenchymal osteogenesis) [7]. The purpose of each component of these building blocks is to replicate the intrinsic properties of autograft reconstructions. However, the strength of scaffolds used in the engineering of bone tissue is not suitable to meet clinical requirements, and restoring the shape of the mandible is difficult.

Some scholars have suggested that the immunogenicity of freeze-dried bone allografts can be removed. Such allografts contain several osteoinductive growth factors (e.g., bone morphogenic proteins [BMPs)). Other non-collagenous proteins in the matrix support the formation of new bone. Allogeneic bone has a similar shape and biological properties. Freeze-dried allogenic bone can be used for scaffolds for bone engineering [8].

Human bone marrow contains stem cells that can differentiate. Mesenchymal stem cells (MSCs) have the potential for multilineage differentiation, and can differentiate into cells with an osteogenic phenotype. Several studies have shown that MSCs can promote osteogenesis in vivo [9]–[11]. These cells can propagate in vitro into the large numbers needed to promote regeneration of injured tissue. MSCs are currently being used in preclinical studies to regenerate bone in patients with massive bone defects.

Here, we employed a tissue-engineering approach to promote the reconstruction of hemi-mandibular defects using mandibular allografts as scaffolds and MSCs as seed cells. The aim of this study was to find out whether this approach can be conducted.

## Materials and Methods

The study was authorized by the Ethics Committee of the General Hospital of the People's Liberation Army (PLA) Beijing China. Beagle dogs were cared for according to the guidelines set by the laboratory Animal Research Center of the General Hospital of the PLA. This study was conducted according to the National Institute of Health (NIH publication No. 85–23, revised 1985) Guideline for the Care and Use of Laboratory Animals.

### Manufacture of freeze-dried allogenic mandibles

**Figure 1:**
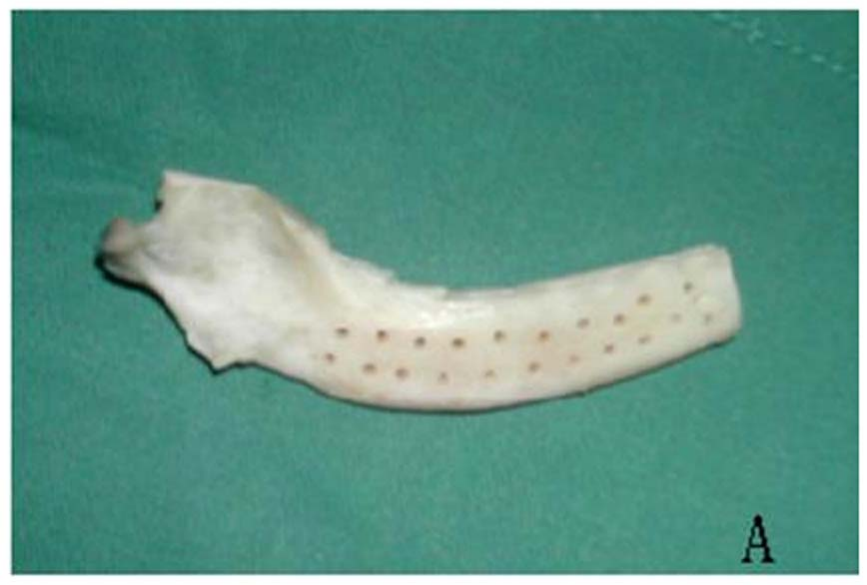
Manufacture of freeze-dried allogenic mandibles.

Mandibles were harvested from 2-year old beagle dogs. Soft tissues and the periosteum were removed and all teeth were extracted. Mandibles were split at the symphysis, and the parts in front of the first premolar were removed. Pores in the bone were then created using a 702-L straight fissure bur (width, 1 mm) at 1-mm intervals until the medullary bone was reached. This procedure was performed under abundant irrigation to allow MSC seeding and to facilitate angiogenesis. Subsequently, mandibles were immersed in 10% hydrogen peroxide for 24 hours at 38°C. The processed mandibles were then incubated in chloroform/methanol (1∶1) for 1 hour at room temperature and then in 0.25% trypsin for 12 hours at 4°C. After this, mandibles were immersed in 0.5% sodium dodecyl sulfate for 6 hours at room temperature. Each processing procedure was followed by extensive washes with distilled water. Mandibles were then freeze-dried, packaged and finally sterilized by ^60^Co gamma-ray irradiation (20Χ10^3^ Gy), and stored at –70°C until implantation (Fig. 1).

### Isolation, cultivation and derivation of MSCs

**Figure 2:**
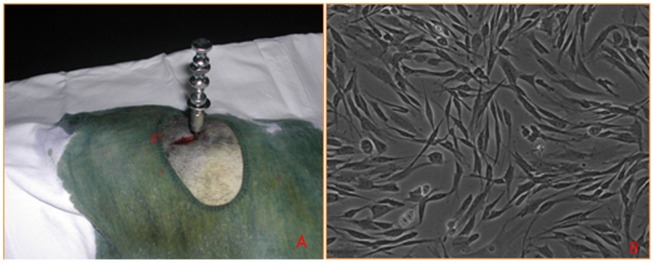
Preparation of the autologous grafts. A: Bone marrow was aspirated from the posterior iliac crest. B: MSCs.

General anesthesia was induced in beagle dogs using an intramuscular injection of xylazine hydrochloride (0.1 ml/kg). From each beagle, a 10-mL sample of bone marrow was aspirated from the posterior iliac crest under local anesthesia (5 ml lidocaine) (Fig. 2A). The sample was transferred to the clean room for cell isolation. The sample was then added to 50 mL of phosphate buffered saline (PBS; Clinimax, Bergisch Gladbach, Germany), loaded onto Lymphodex (Inno-Train, Kronberg, Germany), and centrifuged at 1,500 g for 20 min. Mononuclear cells were then collected and counted using a NucleoCounter NC-100 (ChemoMetec, Allerod, Denmark). The mononuclear cells were washed with PBS and plated at 1×10^6^ cells/cm^2^ in a culture flask with 15 mL alpha-modified minimum Eagle's medium (Gibco, Billings, MT, USA) supplemented with 100 IU/mL penicillin, 100 kg/mL streptomycin (Gibco), and 10% hyclone bovine serum (Thermo Scientific, Thermo Scientific, Waltham, MA, USA). Cultures were expanded through several successive subcultures until a sufficient number of mononuclear cells was achieved (Fig. 2B). Trypan blue staining was used to determine the number of viable cells. For transplantation, approximately 1×10^7^ third-passage cells were suspended in 5 mL of normal saline and transferred to the operating room.

### Injection of MSC-fibrin glue admixture

To load the cells, normal saline was removed from the suspension of MSCs, and the cells were resuspended in 5 mL fibrin glue. Pasteurized fibrin glue (Bolheal, Chemo-Sero-Therapeutic Research Institute, Kumamoto, Japan) was formed by mixing two separate solutions, A and B. Solutions A and B were mixed in a 4∶1 (volume/volume) ratio. Briefly, normal saline was removed from the MSC suspension. The resulting pellet of cell was resuspended in solution A, which consisted of cells (1×10^7^), fibrinogen (80 mg/mL), and fibrin-stabilizing factor XIII (75 units/mL) dissolved in 2.4 mL of plasmin inhibitor aprotinin (1000 kIE/mL). Solution B contained thrombin (250 units) dissolved in 0.6 ml of 40 µM CaCl_2_. Solutions A and B [4∶1 (volume/volume) ratio] were placed in the barrel of a sterilized 5-mL syringe and mixed by inverting the syringe repeatedly. The cell concentration in the admixture was approximately 3×10^6^ cells/mL.

### Experimental design and surgical procedure

**Figure 3:**
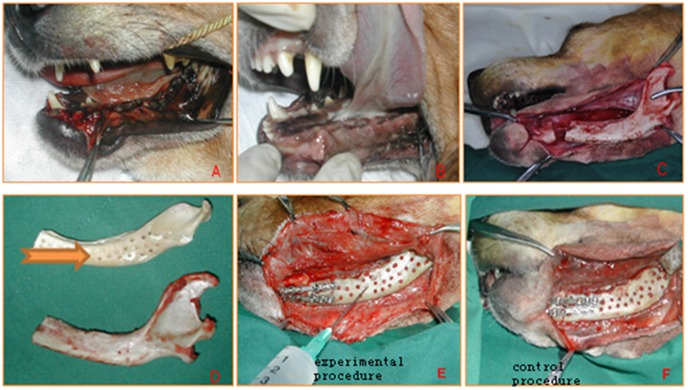
Experimental techniques. A: Extraction of left mandibular teeth. B: Three months after extraction. C: Hemi-mandibular bone was removed to establish a hemi-mandibular defect. D: The shape and size of the allogenic mandibular scaffold was matched (The arrow indicate the allogenic mandibular scaffold). E: In the experimental group, the hemi-mandibular defect was reconstructed using allogenic mandibular scaffold-loaded MSCs. F: In the control group, hemi-mandibular defects were reconstructed using allogenic mandibular scaffolds only.

Thirty healthy adult female beagles (1–2 year; 10.0±3.0 kg) were provided by the Laboratory Animal Center of the Chinese PLA General Hospital (Beijing, China). Surgical methods and animal care conformed to the principles of the Guide for the Care and Use of Laboratory Animals (NIH publication number 85–23, revised 1985). General anesthesia was induced with ketamine hydrochloride (20 mg/kg, im or sc) and maintained with pentobarbital sodium (1–2%, iv). Beagles were prepared for the extraction of left mandibular teeth from the bicuspid tooth to the back molar (Fig. 3A). Three months after extraction, the wounds of the mucosal healed for the next segmental mandibulectomy (Fig. 3B).

All beagles were randomized into two groups: control and experimental. The anesthetized beagle was placed on the operating table in the left lateral position with their limbs in extension and the head aligned with the body and fixed on the left half-face. All surgical interventions were performed by the same surgeon (Changkui L). An incision of length 40–50 mm was made in the left mandible (a lower left paramandibular approach followed the mandible from the angle of the jaw to the chin). A subperiosteal dissection was made to lift a flap, which included skin, from the subcutaneous cellular tissue and mandibular periosteum. In the proximal zone, the mandibular insertion of the masseter and temporalis were separated to expose the outer cortex of the left mandible. Then, the internal cortex was dissected to the adherent gingival mucosa, medial pterygoid muscle, and the lateral pterygoid muscle. Finally, the condyle was dissected from the articular capsule and the inferior alveolar neurovascular bundle was ligated. Using a scroll saw, hemi-mandibular bone was removed from 1 cm of the posterior aspect of the mental foramen to establish a hemi-mandibular defect (Fig. 3C). The defect was replaced with a sterile freeze-dried allograft derived from previously sacrificed beagles and stored at –70°C. Two six-hole titanium plates (3.5-mm wide, 4.0-cm long; Beijing Gemma fly Medical Devices Co., Ltd.) were modeled to fix the graft (Fig. 3E, F). In the experimental group, the hemi-mandibular defect was reconstructed using allogenic mandibular scaffold-loaded autologous MSCs (Fig. 3E). In the control group, hemi-mandibular defects were reconstructed using allogenic mandibular scaffolds only (Fig. 3F). Postoperatively, all beagles were given penicillin (400,000 IU/kg/day, iv) and metronidazole (0.25 mg/day, iv) for 7 days, as required. Beagles were maintained on a soft diet for 12 weeks and subsequently on standard chow until sacrifice.

### Clinical and CT examinations

The activities, responses, food intake, and would healing of surgical sites were observed in all animals. Bone regeneration of the meshes that carried allogenic mandibular scaffold-loaded autologous MSCs and allogenic mandibular scaffolds were examined during transplantation. At 4, 12, 24,and 48 weeks after implantation, four or three beagles of each group were killed by an intravenous overdose of sodium pentobarbitone.

CT scans were conducted before surgery and just after killing using a light-speed 32-slice system (120 kV; 80 mA; rotation time, 0.8 s; slice thickness, 0.2 mm; General Electric, Piscataway, NJ, USA). Three-dimensional reconstruction was conducted to observe the shape, erosion, and calcification of mandibles. Mandibles were excised for further analyses.

### Evaluation of the BMD of mandibles

**Figure 4:**
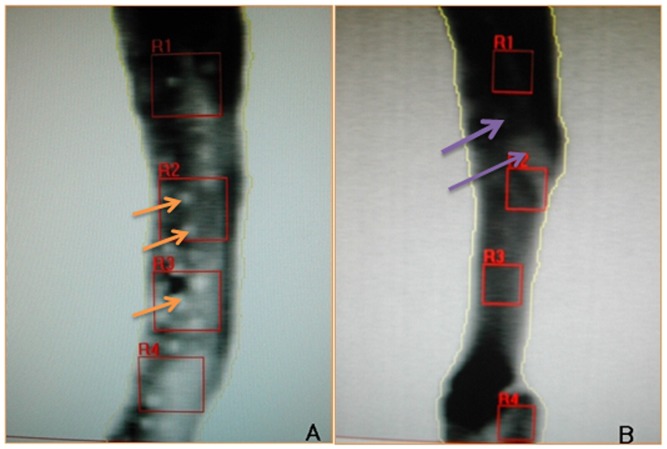
Evaluation of the BMD of mandibles. A: Evaluation of the BMD of control group mandible at 4 weeks after operation. The arrows indicate the pores still can be seen in the control group at this time. B:Evaluation of the BMD of experimental group mandible at 48 weeks after operation. The arrows indicate the pores were almost filled with new bone in the experimental group at this time.

The BMD of the mandibles were evaluated by the 36-XR dual energy x-ray absorptiometry scan (Norland, USA). After scanning the image into the computer, the BDM of 3 × 3-mm size ROI areas were measured by appropriate software. Three times repeated measurements were recorded for each sample and the mean value was calculated (Fig. 4).

### Micro-CT scanning

A micro-CT system was used to quantify the difference of the mandibles in the architecture of the cancellous bone. The prepared specimens of the micro-CT images were placed in a cylindrical cup filled with plastic foam padded to avoid any movement during the scanning procedure. The micro-CT images were obtained using a preclinical cone beam CT scanner (Healthcare Explore Locus SP, GE Medical Systems, Milwaukee, USA) of the Department of Orthopedics of Xijing Hospital. The Explore Locus is a CCD-based camera that acquires data by taking a number of planar images at a regular angular velocity. All adjustable angles were rotated from 180° to 360°. According to the scanning protocol, the images were acquired with the following parameters: (a) 80 kV as the X-ray tube voltage; (b) 80 µA as the anode current; (c) 3000 ms as the exposure time; (d) 1 × 1 as the binning combination; (e) 360° as the rotation angle and 0.4° as the angle increment; and (f) 21 µm as the scanning revolution rate. The micro-architecture of the trabeculae was automatically evaluated using the built-in program of the micro-CT with direct 3D morphometry to reconstruct the 3-D images that consist of an isotropic voxel with a cubic length of 21µm^3^.

A direct, model-independent method was used to quantify various architectural parameters. These parameters included the bone volume fraction (BV/TV), trabecular number (Tb.N), trabecular thickness (Tb.Th), and trabecular separation (Tb.Sp).

### Histological analyses

Mandibular biopsy specimens taken during surgery were fixed in 10% neutral-buffered formalin. After dehydration, specimens were immersed in glycol methacrylate resin (Technovit 7200 VLC; Heraeus-Kulzer, Wehrheim, Germany). After solidification for 24 h, specimens were cut from the longitudinal section of mandibles. Tissues with allogenic mandibular scaffold-loaded autologous MSCs and allogenic mandibular scaffolds were separated for histological examination. Histological preparation was conducted as described previously^9^. Embedded samples were cut into thin sections (40×80 mm) and mounted on glass slides. Two ground sections from each specimen block (300-mm apart) were stained with hematoxylin and eosin (H&E).

### Statistical analyses

Data from radiography and histology are the mean ± SEM and analyzed by one-way ANOVA. Differences between two groups among the six groups were assessed with the Student–Newman–Keuls test. p<0.05 was considered significant.

## Results

### Gross view and CT examinations

All beagles tolerated surgery and were able to eat a normal diet postoperatively. Two beagles from control group and one beagle from the experimental group were eliminated from the analyses because they developed postoperative wound infections.

**Figure 5:**
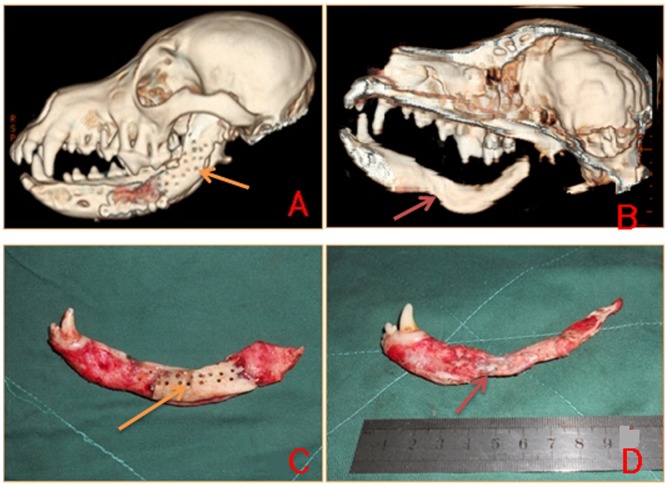
The shape of the mandibles was observed by gross inspection and three-dimensional CT reconstruction. A: The shape of the mandibles as observed by three-dimensional CT reconstruction showed no resorption of the allogenic mandibular scaffolds in the control group at 48 weeks. The arrow indicate the pores still can be seen in the control group at this time. B: Resorption was observed in the experimental group at 48 weeks. The arrow indicate the resorption. C: The pores still can be seen in the control group at 48 weeks. The arrow indicate the pores still can be seen in the control group at this time. D: The pores were filled with new bone in the experimental group at 48 weeks. The arrow indicate the resorption.

The shape of the mandibles was observed by three-dimensional CT reconstruction and, after dissection, showed resorption of allogenic mandibular scaffolds in the experimental group at 4 weeks, but such resorption was not observed in the control group. At 12 weeks, new bone had formed in an irregular fashion on allogenic mandibular scaffolds in the experimental group. In the control group, new bone formation and scaffold absorption was not significant at 12 weeks. The shape of mandibles in the beagles of the two groups returned to almost normal at 48 weeks after surgery, but the pores were filled with new bone in the experimental group (Fig. 5).

### Mandibular BMD

Twelve weeks after transplantation, the experimental group BMD was significantly higher than in the control group (p<0.05). With the progression of time, the BMD of the experimental group and the control group both increased, but at a significantly higher rate in the experimental group (Table 1).

**Table 1:** The BMD of the experimental group and the control group (g/m^2^, *x* ± s).

	4 wk	12 wk	24 wk	48 wk
Experimental group	0.246±0.034	0.434±0.52	0.512±0.035	0.554±0.056
Control group	0.295±0.042	0.298±0.43	0.312±0.053	0.391±0.047
P-value	>0.05	<0.05	<0.05	<0.05

### Micro-CT findings

The mean, standard deviation and range for each of the micro-architectural parameters from the allogenic mandibular scaffolds of 30 beagles were tabulated, and the bone micro-architecture parameters of the allograft bone at 1cm of the proximal end were analyzed in 30 beagles in both the experimental and control groups (Table 2). The mean D-value of all micro-architectural parameters were significantly different between the groups (p < 0.05).

**Table 2:** Micro-architectural parameters of the experimental group and the control group (aggregate of all samples).

Parameters	Tb.N (/mm)	Tb.Th (mm)	Tb.Sp (mm)	BV/TV(%)
Experimental group	2.34±0.47	0.13±0.04	0.47±0.22	0.24±2.2
Control group	2.93±0.13	0.15±0.02	0.31±0.04	0.48±0.6
P-value	<0.05	<0.05	<0.05	<0.05

### Histological findings

**Figure 6:**
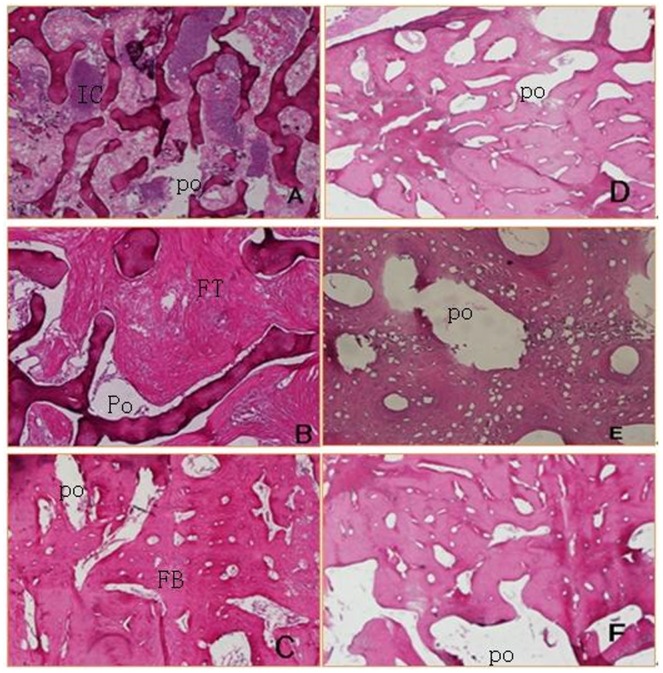
Histological analyses. A: Many inflammatory cells filled in the allogenic mandibular scaffold in the experimental group 4 weeks postoperatively. B: The pores were filled with fibrous tissue in the experimental group at 12 weeks after surgery. C: Most of the transplanted allogeneic bone was replaced by new bone (fibrous ossification) in the experimental group at 48weeks after the reconstruction. D: Allogeneic bone edges showed less absorption in the control group at 4 weeks after surgery. E: The pores could still be seen in the control group at 12 weeks after surgery. F: A small part of the allogenic bone graft was replaced by new bone in the control group at 48 weeks after reconstruction.(IC: inflammatory cell. Po: pore. FB: fibrous ossification. FT: fibrous tissue.)

A small amount of trabecular bone growth towards allogenic bone was observed in the experimental group 4 weeks after surgery. Cortical allograft bone edges showed lacunar absorption, and a small amount of new bone was formed. The highest proportion of cells filling the allogenic mandibular scaffold were inflammatory (Fig. 6A). However, in the control group, allogeneic bone edges showed less absorption, and virtually no new trabecular bone formed between autologous bone and allograft bone 1 month after surgery (Fig. 6D).

Twelve weeks after surgery, trabecular bone was observed to connect autologous and allograft bone in the experimental group scaffolds. Osteoblast progenitor cells and osteoblasts were observed to grow towards the inside of allogenic mandibular scaffolds at the edge of the allogeneic bone. Allogeneic bone was partially absorbed, the Haversian canal expanded, and fibrous tissues grew into allogenic mandibular scaffolds (Fig. 6B). Resorption of allogeneic bone was not clearly observed in the control group and there fewer fibrous tissues grew towards the inside of allogenic mandibular scaffolds than observed in the experimental group (Fig. 6E).

Forty-eight weeks after reconstruction, the new bone graft in the experimental group was smaller than the original, but almost all transplanted allogeneic bone was replaced by new bone, although most of it was fibrous tissues (Fig. 6C). Allogeneic bone was not absorbed in the control group, and it maintained its original shape. A small part of allogenic bone was replaced by new bone or fibrous tissues (Fig. 6F).

## Discussion

This study demonstrated the successful reconstruction of beagle hemi-mandibular defects with allogenic mandibular scaffolds and autologous mesenchymal stem cells. The engineering of bone tissue requires three factors: scaffolds, seed cells, and growth factors. Studies have shown that bone induction is the main healing method after bone allografting. Bone induction is that allogenic bone in the form of scaffolds can induce stem cells surrounding the bone to be converted into osteoblasts and gradually result in osteogenesis. There are several advantages in using allogenic bone as scaffolds for the reconstruction of mandibular defects. These materials have structural similarities to host bone and are available in various shapes and sizes for mandibular defects. Also, as with autologous bone grafts, they can be incorporated into surrounding bone over time through “creeping substitution”. Most importantly, obtaining allografts does not require killing host structures. Using an allogenic mandible as a scaffold for tissue engineering can be monitored with simple panoramic imaging as well as CT because of its similar density and porosity to natural bone.

Regarding seed cells, human bone marrow contains stem cells that can differentiate. Mesenchymal stem cells (MSCs) have the potential for multilineage differentiation, and can differentiate into cells with an osteogenic phenotype. Several studies have shown that MSCs can promote osteogenesis in vivo [9]–[11]. These cells can propagate in vitro into the large numbers needed to promote the regeneration of injured tissue.

The study has showed that the freeze-dried bone contain many growth factors such as BMP[12]. BMPs have unique osteoinductive proprieties. They act at an early stage and maintain both bone and cartilage formation, boosting the MSCs among cells with bone- and cartilage-forming capacity.

However, two factors influence the formation of bone tissue: the local milieu and the maintenance of stem cells in situ. Immunogenicity may be removed from freeze-dried bone allografts but cortical bone remains dense. This scenario is not conducive to permitting cells and nutrients to penetrate bone. Hence, we created many pores on the bone allograft scaffolds to permit the penetration of cells and nutrients. Fibrin glue was used in this study to maintain the position of the stem cells. Several studies have suggested that fibrin glue can be used for cell delivery because it is a biocompatible and biodegradable tissue adhesive that stabilizes seeded cells and provides an equally distributed population of cells throughout the carrier. Authors have demonstrated that fibrin glue does not inhibit the proliferation of bone-marrow MSCs [13]–[15].

In this study, allogeneic bone began to absorb 1 month after transplantation in the experimental group. Absorption was significant 3 months after transplantation and new bone was formed at this time. The size of new bone was smaller than that of the original bone allograft scaffold, and almost all the bone allograft scaffold was replaced by new bone 1 year after transplantation. However, in the control group, the bone allograft was not absorbed 1 year after transplantation. The speed of the formation of new bone was slower than that observed in the experimental group. Approximately 30% of allogenic bone was replaced by new bone. It is possible that more dense bone was formed at the cortical bone, and therefore angiogenesis was slower.

Resorption of allogeneic bone in the experimental group was significantly greater (especially in the condyle), and formation of new bone faster, than that seen in the control group. These findings demonstrated that the mechanism of healing of bone allografts changed when bone marrow-derived MSCs were loaded onto the scaffolds. It is possible that the addition of cells with high osteoblastic potential could enhance the bone appositional phase from the early stages of remodeling. Nevertheless, investigation of the specific underlying mechanism merits further study.

However, histological analysis has shown that although almost all transplanted allogeneic bone was replaced by new bone in the experimental group, most of it was fibrous ossification. MSCs have the potential for multilineage differentiation into osteoblasts, chondrocytes, adipocytes, myocytes, cardiomyocytes, and neurons, amongst others. They can differentiate into osteoblasts in the presence of osteoinductive factors or an osteogenic environment. In this study, we used scaffolds and seed cells, and did not add growth factors during the reconstruction of hemi-mandibular defects. Although the freeze-dried bone contain many growth factors such as BMP, the presence of growth factors in the freeze-dried bone was very limited. These limited growth factors could not have induced MSCs to differentiate into osteoblasts. It is possible reason that most of the “new bone” was fibrous ossification. In future studies, we will add growth factors such as BMP to assess whether more optimal results can be gained.

In summary, we have demonstrated that tissue-engineered bone could be created using bone allograft scaffold-loaded autologous marrow MSCs. MSCs accelerate the speed of absorption of bone allografts and ossification. The major drawback is infection, as two beagles from control group and one beagle from experimental group had postoperative wound infections, and future studies will be needed to determine ways to reduce the rate of this complication.
